# Gut microbiota is associated with response to ^131^I therapy in patients with papillary thyroid carcinoma

**DOI:** 10.1007/s00259-022-06072-5

**Published:** 2022-12-13

**Authors:** Lei Zheng, Linjing Zhang, Li Tang, Dingde Huang, Deng Pan, Wei Guo, Song He, Yong Huang, Yu Chen, Xu Xiao, Bo Tang, Jing Chen

**Affiliations:** 1 Nuclear Medicine Department, Southwest Hospital (the First Affiliated Hospital), Third Military Medical University (Army Medical University), Chongqing, China; 2 Department of Gastroenterology, Xinqiao Hospital (the Second Affiliated Hospital), Third Military Medical University, (Army Medical University), Chongqing, China; 3 State Key Laboratory of Trauma, Burns and Combined Injury of China, Institute of Burn Research, Southwest Hospital (the First Affiliated Hospital), Third Military Medical University, (Army Medical University), Gao Tan Yan Street, Chongqing, 400038 China

**Keywords:** Gut microbiota, Radioactive iodine therapy, ^131^I therapy, 16s rRNA, Predictive model, ^131^I response

## Abstract

**Purpose:**

Radioactive iodine (^131^I) therapy is a conventional post-surgery treatment widely used for papillary thyroid carcinoma (PTC). Since ^131^I is orally administered, we hypothesize that it may affect gut microbiome. This study aims to investigate alterations of intestinal microbiome caused by ^131^I therapy in PTC patients and explore its association with response to ^131^I therapy.

**Methods:**

Fecal samples of 60 PTC patients pre- and post-^131^I therapy were collected to characterize the ^131^I therapy-induced gut microbiota alterations using 16S rRNA gene sequencing. According to the inclusion criteria, sequence data of 40 out of the 60 patients, divided into excellent response (ER) group and non-excellent response (NER) group, were recruited to investigate the possible connection between gut microbiota and response to ^131^I therapy. Multivariate binary logistic regression was employed to construct a predictive model for response to ^131^I therapy.

**Results:**

Microbial richness, diversity, and composition were tremendously altered by ^131^I therapy. A significant decline of Firmicutes to Bacteroides (F/B) ratio was observed post-^131^I therapy. ^131^I therapy also led to changes of gut microbiome-related metabolic pathways. Discrepancies in β diversity were found between ER and NER groups both pre- and post-^131^I therapy. Furthermore, a predictive model for response to ^131^I therapy with a *p* value of 0.003 and an overall percentage correct of 80.0% was established, with three variables including lymph node metastasis, relative abundance of g_Bifidobacterium and g_Dorea. Among them, g_Dorea was identified to be an in independent predictor of response to ^131^I therapy (*p* = 0.04).

**Conclusion:**

For the first time, the present study demonstrates the gut microbial dysbiosis caused by ^131^I therapy in post-surgery PTC patients and reveals a previously undefined role of gut microbiome as predictor for ^131^I ablation response. G_Dorea and g_Bifidobacterium may be potential targets for clinical intervention to improve response to ^131^I in post-operative PTC patients.

**Trial registration:**

ChiCTR2100048000. Registered 28 June 2021.

## Introduction

Thyroid carcinoma (TC) is the commonest endocrine malignancy, the incidence of which is still increasing worldwide. Differentiated thyroid cancer (DTC) takes up over 90% of all TC, among which, papillary thyroid carcinoma (PTC) is the foremost histopathologic type, taking up more than 85% of TC [1].

Radioactive iodine (^131^I) therapy is the mainstay of treatment for PTC after surgery [2], which has been used for nearly 80 years and still plays a central role in the management of PTC today [3]. However, response to ^131^I therapy varies among patients. Distinct responses to ^131^I determine different subsequent clinical strategies and prognosis. According to the 2015 American Thyroid Association Management Guidelines for Adult Patients with Thyroid Nodules and Differentiated Thyroid Cancer [4], responses to ^131^I ablation can be categorized as excellent response (ER) or non-excellent response (NER). PTC patients with a negative imaging, negative thyroglobulin antibody (TgAb) and either suppressed thyroglobulin (Tg) < 0.2 ng/ml or thyroid stimulating hormone (TSH) stimulated Tg (sTg) < 1 ng/ml are evaluated as ER. Those who do not achieve these standards are defined as NER. ER to ^131^I indicates clinical cure of PTC, whereas patients with NER may require another course of ^131^I therapy. Hence, the need for a reliable tool predicting therapeutic response to ^131^I ahead of ^131^I therapy is vital and urgent, but has not been fulfilled yet.

^131^I is an orally administered radioactive nuclide used for internal-radiation therapy to treat PTC. After application, ^131^I stays and accumulates in the gastro-intestinal tract, thus is very likely to affect gut microbiome. Gut microbiota and its vital role in various diseases have drawn more and more attention. Accumulating evidences have indicated that gut microbiota plays a part in the pathophysiology of cancer [5]. Recently, a close connection between gut microbiome and thyroid carcinoma has been implicated [6]. Another study finds out that gut microbiome is tremendously altered in thyroid carcinoma patients compared to control subjects [7]. Thyroid function is influenced by gut microbiota [8]. Moreover, gut microbiota is also reported to be associated with radiation sensitivity and radiation-related toxicities [9–11]. Evidences from animal models have also shown that gut microbiome composition may predict radiation injury [9].

However, the alterations of gut microbiota after ^131^I therapy and the significance have not been elucidated yet. Whether difference in gut microbiome is related to distinct responses to ^131^I remains elusive. Can one or several gut genera be used as predictors of ^131^I response in PTC patients before the therapy is still unknown.

Given the paucity of gut microbiome studies in post operative PTC patients receiving ^131^I therapy, we reported the present prospective study.

## Materials and methods

### Study participants

This study was performed in agreement with the principles of the Declaration of Helsinki. Approval was granted by the Ethics Committee of the First Affiliated Hospital of Army Medical University (Third Military Medical University), China (Date Oct. 16th, 2020/No. KY2020214). The authors affirmed that all participants provided informed consents for clinical data and bio-sample use and publication of their basic characteristics and 16s rRNA sequence results of their stools.

All patients included in this study were from the southwest region of China (including Chongqing, Sichuan, and Guizhou), where the climate and residents’ eating habits were similar. ^99m^TcO4^−^ thyroid imaging was negative before the ^131^I therapy on every single participant. Forty-eight hours after taking ^131^I orally at a dose of 150 mCi (5.55 GBq), a very small part of thyroid tissue was displayed in all of the patients by post radioactive iodine therapy whole-body scanning (RxWBS), with no significant difference in the amount of residual thyroid tissue among the patients. Serum TSH concentration of each participant before ^131^I therapy was over 30 mIU/L.

### Self-controlled study cohort, recruitment of subjects, procedures of ^131^I therapy, and sampling

The inclusion criteria for TC patients were as follows: (1) patients were diagnosed as PTC; (2) patients had undergone complete thyroid resection procedure; (3) patients were scheduled to receive ^131^I therapy for the first time; (4) patients were willing to participate in this study and signed the informed consent forms; (5) patients promised to voluntarily accept and comply with this experimental protocol.

The exclusion criteria were as follows: (1) patients with known history of any other cancer besides PTC; (2) patients who had undergone prior ^131^I therapy or other radiotherapy; (3) patients with notable gastrointestinal disorder; (4) patients with known history of gastrointestinal surgery; (5) patients with long-term use of any probiotics or antibiotics, non-steroid anti-inflammatory drugs or proton pump inhibitors; (6) patients with an age < 18 years. Finally, a total of 60 subjects from the nuclear medicine department of the First Affiliated Hospital of the Army Medical University (Third Military Medical University), China, between October 2020 and March 2021, who fulfilled the inclusion criteria and provided the fecal and blood samples, were included in the self-controlled study to explore microbiota changes induced by ^131^I therapy.

Baseline characteristics and clinical parameters are listed in Table 1. Patients were given iodine-free diet for 4 weeks and underwent 3 weeks of Euthyrox withdrawal before each sampling. Two sequential peripheral blood and fecal samples were collected from patients at time points 1–2 days before and 5 months after ^131^I administration, respectively. Since the patients recruited in this study are either high-risk PTC, or intermediate-risk PTC with aggressive histology or with unexplained elevated sTg levels (sTg > 10 ng/ml) or with both, ^131^I was administered orally at a dose of 150 mCi (5.55 GBq) for adjuvant therapy. All samples were aliquoted and stored at − 80 °C for further use. TSH and sTg levels in the blood samples were assayed. DNA extraction and 16S rRNA gene sequencing were performed using the fecal samples.

**Table 1:** Demographic and clinical characteristics of participants in self-controlled study

Baseline characteristics	Participants (*n* = 60)
Age, years: mean ± SD	40.02 ± 10.23
Gender: *N* (%)	Male: 19 (31.7%) Female: 41 (68.3%)
Risk level: N(%)	Low: 0 (0.0%)
	Medium: 49 (81.7%)
	High: 11 (18.3%)
Pathologic stage post-surgery: *N* (%)	I: 54 (90.0%)
	II: 4 (6.7%)
	III: 2 (3.3%)
	IV: 0 (0.0%)
Maximum tumor diameter, cm: mean ± SD	1.38 ± 0.82
Local invasion by pathology: *N* (%)	Without: 43 (71.7%)
	With: 17 (28.3%)
Lymph node metastasis by RxWBS^a^: *N* (%)	Without: 49 (81.7%)
	With: 11 (18.3%)
Distant metastasis by RxWBS: *N* (%)	Without: 59 (98.3%)
	With: 1 (1.7%)
sTg^b^ or TgAb^c^ level:	
sTg < 1 ng/ml: *N* (%)	25 (41.7%)
sTg 1–10 ng/ml: *N* (%)	22 (36.7%)
sTg > 10 ng/ml or rising TgAb level: *N* (%)	13 (21.6%)

^a^RxWBS, post radioactive iodine therapy whole-body scanning

^b^sTg, TSH-stimulated thyroglobulin

^c^TgAb, thyroglobulin antibody

### Patient demographics and clinical parameters of ^131^I ER and NER patients

According to the definition of ER demonstrated previously, before ^131^I ablation, 20 out of the 60 patients already fulfilled all other criteria of ER (no metastases observed, sTg < 1 ng/ml and negative TgAb) except for very small amounts of thyroid remnant imaging as shown by RxWBS, indicating that after ^131^I ablation, it was very likely that these 20 patients would be classified as ER according to both our experiences and literatures [12]. As a matter of fact, after ^131^I ablation, they were actually evaluated as ER in this study. Thus, in order to reduce false-positive rate, these 20 patients were excluded from this part of study.

The rest 40 participants were divided into ^131^I ER group (24 cases) and NER group (16 cases) based on their assessment post ^131^I ablation according to the guidelines. Gut microbiota of the two groups both 1–2 days pre-treatment of ^131^I (Pre-) and 5 months post-treatment (Post-) were analyzed and compared.

### DNA extraction and 16S rRNA sequencing analysis

Fecal DNA extraction was performed using CTAB method. The V3–V4 region of the bacterial 16S rRNA gene was amplified by Phusion® High-Fidelity PCR Master Mix (New England Biolabs, New England) using specific primers (515F-806R) with a barcode. PCR product purification was carried out with Qiagen Gel Extraction Kit (Qiagen, Germany). TruSeq® DNA PCR-Free Sample Preparation Kit (Illumina, USA) was used to generate sequencing libraries. The libraries were sequenced using the Illumina NovaSeq 6000 platform (Novogene Company, China). We took advantage of Microbial Ecology 2 (QIIME2, version 2020. 2) [13] platform to process the sequencing data in a conda environment. In brief, the V3–V4 primers of paired-end fastq format sequence files were trimmed using cutadapt 3.1 [14]. Next, trimmed fastq files were imported into QIIME2. After the DADA2 denoising, a feature table listing sequence number of all samples and features was analyzed. Samples with less than 8000 sampling depth were excluded. All samples were normalized to the same depth. After rarefaction, alpha diversity and beta diversity were calculated respectively. Then, the results of the principal coordinate analysis (PcoA analysis) was calculated. Taxonomic composition of the samples were also displayed. The discrepantly abundant bacterial taxa between two groups were analyzed and displayed by linear discriminant analysis effect size (LEfSe) [15].

### Establishment of a response-prediction model for ^131^I therapy

Preliminary covariates were discriminatory gut taxa between ER and NER pre-^131^I therapy and some possibly associated clinical factors, including relative abundance of Tannerellacea, g_Parabacteroides, g_Dorea, g_Bifidobacterium, f_Bifidobacteriaceae, o_Bifidobacteriales, f_Erysipelotrichaceae, c_Erysipelotrichia, o_Erysipelotrichales, age, gender, risk level, pathologic stage, maximum tumor diameter, local invasion, lymph node metastasis, distant metastasis, sTg or TgAb level, and Firmicutes/Bacteroidetes ratio. Variable screening was carried out by determination method based on characteristic root. In short, 5 variables, including f_Tannerellaceae, f_Bifidobacteriaceae, o_Bifido-bacteriales, c_Erysipelot-richi, and o_Erysipelotrichales, with multicollinearity were eliminated from the model. It was validated that there was no multicollinearity among the left variables post screening by both the characteristic root determination method and calculation of the ranks of matrixes. The left variables, including age, gender, risk level, pathologic stage, maximum tumor diameter, local invasion, lymph node metastasis, distant metastasis, sTg or TgAb level, Firmicutes/Bacteroidetes ratio, relative abundance of g_Parabacteroides, g_Dorea, g_Bifidobacterium, and f_Erysipelotrichaceae, were analyzed by multivariate analysis using binary logistic regression with backward stepwise to establish the predictive model. After 12 rounds of conditional backward stepwise removal of covariates with non-significant predictive effect, ultimately 3 variables, consisting of lymph node metastasis, relative abundance of g_Bifidobacterium and g_Dorea, were screened out as the optimal set of characteristic variables to predict patients’ response to ^131^I therapy. The statistical software used was IBM SPSS Statistics 26.0.

### Bioinformatics and statistical analysis

Alpha (*α*) diversity was evaluated by a set of indexes, including Shannon, Simpson, ACE, and CHAO1, while beta (*β*) diversity was assessed using Bray–Curtis distance-based non-metric multidimensional scaling (NMDS) analysis, supervised partial least squares-discriminant analysis (PLS-DA), PCoA based on unweighted unifrac distance matrix or based on Jaccard index. Taxa bar plot and LEfSe analysis were performed to distinguish discrepant abundant genera between the two groups. The predicted metabolic functional differences of gut microbiota between the two groups were compared using picrust2 to identify differentially involved Kyoto Encyclopedia of Genes and Genomes (KEGG) pathways.

The normality of distribution was determined using Shapiro–Wilk test. Homogeneity of variance was tested by *F* test. Comparison of baseline characters was performed by *χ*^2^ or Student’s *t*-test. Paired *t*-test was used in the statistical analysis of self-controlled study. Significance between two groups was determined by Student’s *t*-test or Wilcoxon-Rank test. *P* values < 0.05 were considered significant.

## Results

### Study cohorts and clinical parameters of self-controlled study

Sixty post-operative PTC patients, who were planning for ^131^I therapy, were recruited in the self-controlled study to explore microbiota changes due to ^131^I therapy. Baseline characters are listed in Table 1.

### Microbial richness, diversity, and composition alteration after ^131^I therapy

**Fig. 1:**
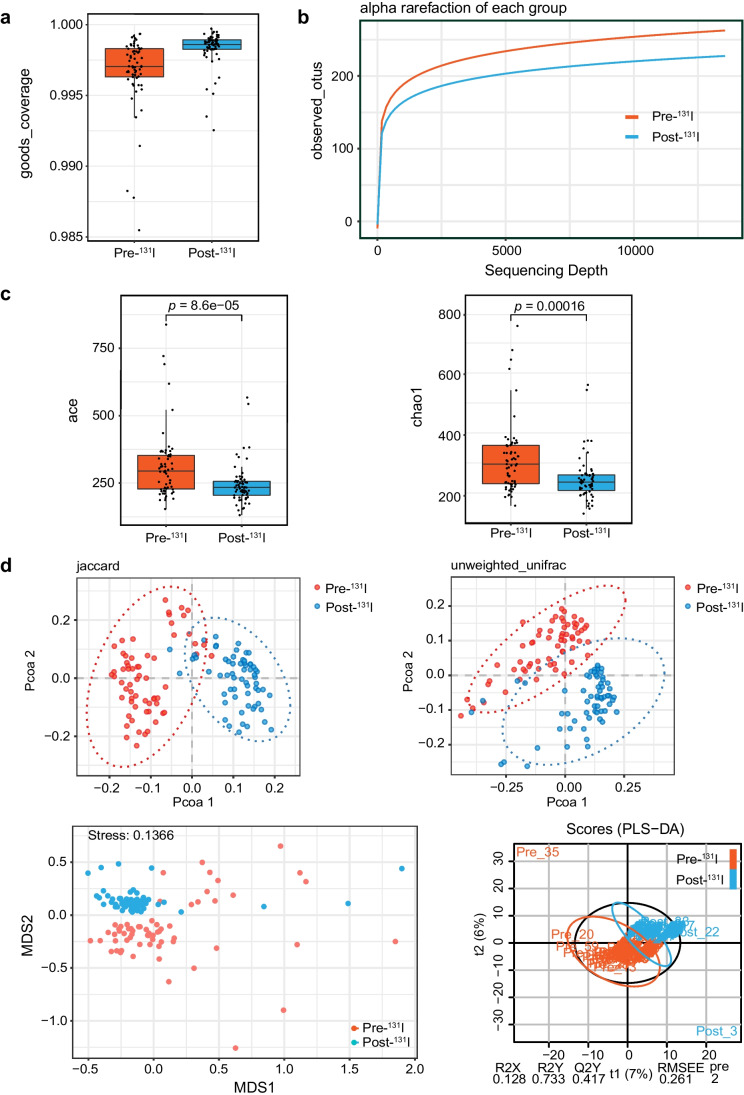
Microbial richness and diversity alterations post-^131^I therapy. Pre-^131^I: 1–2 days before ^131^I administration; Post-^131^I: 5 months after.^131^I administration. **a** Goods_coverage indexes of two groups. **b** Alpha rarefaction of each group indicating sequencing depth. **c** Ace and Chao1 indexes of the taxonomic α diversities. Ace: paired *t*-test, *p* = 8.6e-05. Chao1: paired *t*-test, *p* = 0.00016. **d** PCoA plot, NMDS, and PLS-DA indexes of the *β* diversity (paired *t*-test, PCoA unweighted index: *r* = 0.2037, *p* = 0.001; PCoA Jaccard index: *r* = 0.2813, *p* = 0.001; NMDS: stress = 0.1366; PLS-DA: R2X = 0.128, R2Y = 0.733, Q2Y = 0.417)

From the 60 self-controlled fecal samples, 7,540,436 high-quality sequences were used (average 59,845 per sample). The total number of ASV was 9583 at 99% similarity level. The Good’s coverage of each group was over 99% (Fig. 1a). In addition, alpha Rarefaction curve in each group is nearly smooth with a sufficient amount of sequencing data (Fig. 1b), indicating that the sequence depth represented the majority of gut bacteria in the samples.

The taxonomic *α* diversity indicated by ACE (Abundance-based Coverage Estimator) richness (*p* = 8.6e − 05) and Chao1 (*p* = 0.00016) indexes displayed significantly lower community richness and diversity in the fecal microbiota after ^131^I therapy (Fig. 1c).

Shifts in β diversity (composition and structure) from pre-^131^I therapy to post-^131^I therapy were also observed by PCoA based on unweighted unifrac distance matrix and Jaccard index, NMDS analysis, and PLS-DA (Fig. 1d). All results indicated that the samples within each group were clustered together, while the samples between groups were separated, illustrating prominent differences in bacterial structure pre- and post-^131^I therapy (PCoA unweighted index: *r* = 0.2037, *p* = 0.001; PCoA Jaccard index: *r* = 0.2813, *p* = 0.001; NMDS: stress = 0.1366; PLS-DA: R2X = 0.128, R2Y = 0.733, Q2Y = 0.417).

**Fig. 2:**
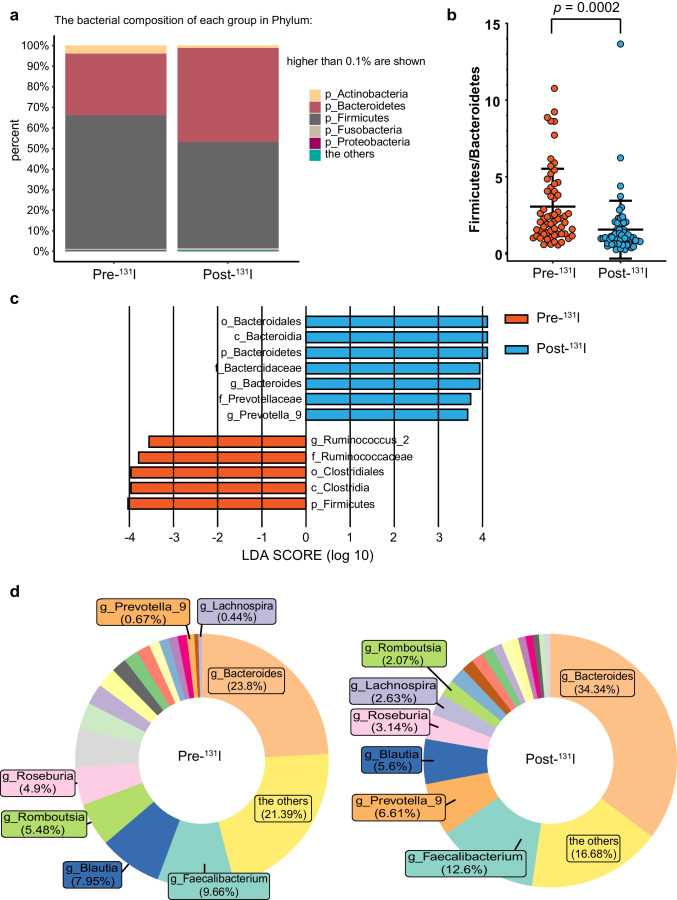
Microbial composition alterations post-^131^I therapy. **a** Taxa bar plot illustrating the bacterial composition of each group in Phylum. **b** Firmicutes to Bacteroides (F/B) ratio of the two groups. Paired *t*-test. *p* = 0.0002. **c** Linear discriminant analysis effect size (LEfSe) analysis indicating differentially abundant bacterial taxa with a LDA score over 3.5 between the two groups. **d** Pie plot showing genus-level distribution of fecal microbiota

Taxa bar plot showed the bacterial composition of each group in Phylum (Fig. 2a). The most pronounced differences were decrease of Firmicutes and increase of Bacteroidetes, consequently leading to a significant decline in the Firmicutes to Bacteroides (F/B) ratio (*p* = 0.0002) after treatment (Fig. 2b), suggesting dysbiosis following ^131^I therapy. LEfSe analysis illustrated remarkably different microbes between the two groups with a LDA score over 3.5 (Fig. 2c), with notable increments in relative abundance of f_Bacteroidaceae and f_Prevotellaceae post-^131^I therapy, and reverse trend in f_Ruminococcaceae. Genus-level distribution alterations of fecal microbiota was demonstrated by pie plot (Fig. 2d), showing an overall pattern of an increase in “pathogenic microbiota,” for example, Bacteroides, Prevotella_9, and a decrease in “beneficial microbiota,” including Roseburia and Blautia.

### Predicted functional changes of microbiome induced by ^131^I therapy

**Fig. 3:**
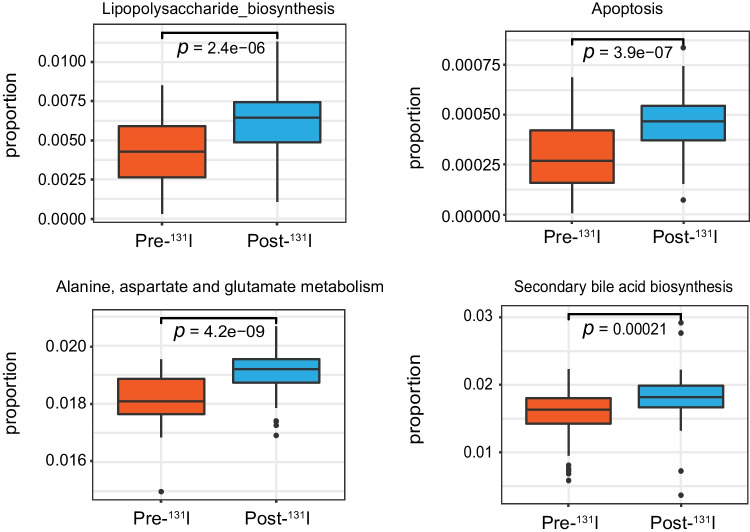
Significantly altered KEGG pathways of gut microbiome post-^131^I therapy

We further investigated the predicted functional alterations in gut microbiota owing to ^131^I therapy. Analysis of KEGG pathways showed that compared with microbiome before ^131^I therapy, after ^131^I therapy, pathways involved in lipopolysaccharide biosynthesis, apoptosis, alanine, aspartate, and glutamate metabolism, etc., were markedly altered (Fig. 3).

### Study cohorts and clinical parameters of ^131^I ER and NER patients

After identifying the impact of ^131^I therapy on gut microbiota, we then explored whether microbiome dysbiosis would be correlated with therapeutic response to ^131^I therapy. After aforementioned exclusion of 20 patients, 40 participants were included and classified into ^131^I ER group or NER group. Patients’ demographic features and clinical parameters are shown in Table 2.

**Table 2:** Demographic and clinical characteristics of participants in the curative effect-controlled study

Baseline characteristics	Participants (*n* = 40)		Comparison between groups (*p* value)
	Excellent response (ER)	Non-excellent response (NER)	
Case: *N* (%)	24 (60%)	16 (40%)	
Age, years: mean ± SD	38.67 ± 9.7	35.63 ± 9.0	0.324
Gender: *N* (%)	Male: 8 (33.3%) Female: 16 (66.7%)	Male: 8 (50%) Female: 8 (50%)	0.234
Risk level: *N* (%)	Medium: 20 (83.3%) High: 4 (16.7%)	Medium: 14 (87.5%) High: 2 (12.5%)	0.718
Pathologic stage post-surgery: *N* (%)	I: 22 (91.6) II: 1 (4.2%) III: 1 (4.2%)	I: 16 (100%) II: 0 (0%) III: 0 (0%)	1.000
Maximum tumor diameter, cm: mean ± SD	1.42 ± 0.95	1.48 ± 0.80	0.852
Local invasion by pathology: *N* (%)	Without: 18 (75%) With: 6 (25%)	13 (81.3%) 3 (18.7%)	0.717
Lymph node metastasis by RxWBS^a^: *N* (%)	Without:15 (62.5%) With: 9 (37.5%)	14 (87.5%) 2 (12.5%)	0.148
Distant metastasis by RxWBS: *N* (%)	Without: 23 (95.8%) With: 1 (4.2%)	16 (100%) 0 (0%)	1.000
sTg^b^ or TgAb^c^ level:			
sTg < 1 ng/ml: *N* (%) sTg 1–10 ng/ml: *N* (%) sTg > 10 ng/ml or rising TgAb level: *N* (%)	4 (16.7%) 15 (62.5%) 5 (20.8%)	1 (6.3%) 7 (43.7%) 8 (50%)	0.186

^a^RxWBS, post radioactive iodine therapy whole-body scanning

^b^sTg, TSH-stimulated thyroglobulin

^c^TgAb, thyroglobulin antibody

### Gut microbiota richness, diversity, and composition differences between ^131^I ER and NER group pre- and post-^131^I therapy

**Fig. 4:**
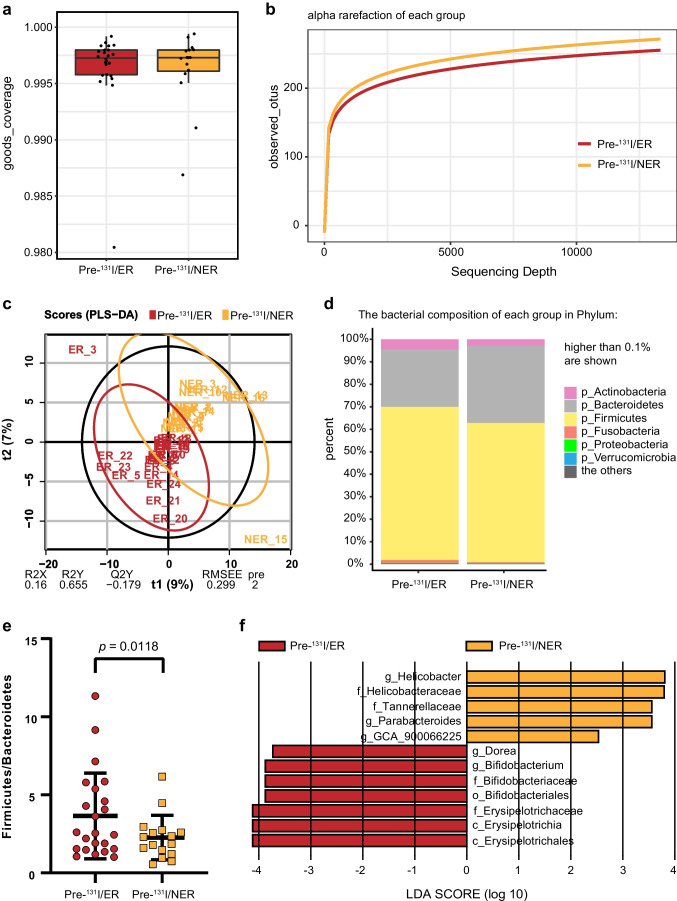
Significant differences in microbial richness, diversity, and composition between excellent response (ER) and non-excellent response (NER) groups 1–2 days before ^131^I therapy. ER: excellent response to ^131^I therapy; NER: non-excellent response to ^131^I therapy. **a** Goods_coverage indexes of two groups. **b** Alpha rarefaction of each group indicating sequencing depth. **c** PLS-DA-plot of the two groups. **d** Taxa bar plot indicating the bacterial composition of each group in Phylum. **e** Firmicutes to Bacteroides (F/B) ratio of the two groups (*t*-test, *p* = 0.0002). **f** LEfSe analysis showing significantly different gut flora with a LDA score over 2 between the two groups

At 1–2 days prior to ^131^I radiotherapy, from the 40 participants’ samples, 2,268,985 high-quality sequences were used (average 56,725 per sample). The total number of ASV was 4958 at 99% similarity level. The Good’s coverage (over 99%) (Fig. 4a) and the observed species rarefaction curve (Fig. 4b) were used to characterize good sequencing depths. The microbiota of the two groups showed no significant difference in *α* diversity (data not shown). *β* diversity analysis represented by PLS-DA-plot illustrated that the microbiome samples were clustered by group (Fig. 4c; R2X = 0.16, R2Y = 0.655, Q2Y =  − 0.179). Different bacterial composition of each group in Phylum was shown in taxa bar plot (Fig. 4d). A prominent decrease of F/B ratio was observed in the NER group as compared to ER group (Fig. 4e; *p* = 0.0118). LEfSe analysis illustrated substantially differently abundant bacterial genera, including Parabacteroides, Dorea, and Bifidobacterium and microbial family, such as Erysipelotrichaceae with a LDA score over 2.0 between the two groups (Fig. 4f).

**Fig. 5:**
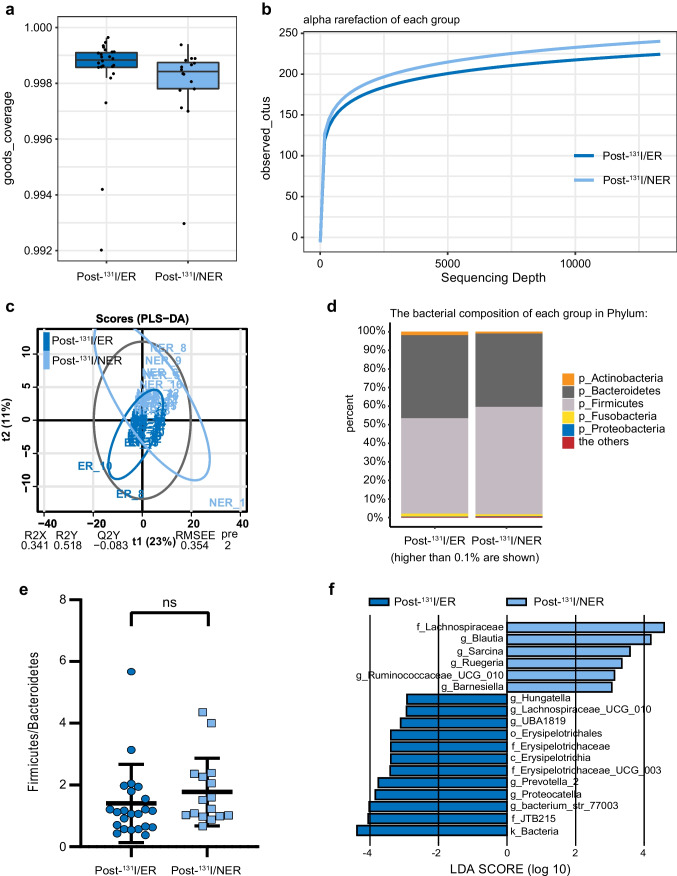
Significant differences in microbial richness, diversity, and composition between excellent response (ER) and non-excellent response (NER) groups 5 months after ^131^I therapy. **a** Goods_coverage indexes of two groups. **b** Alpha rarefaction of each group indicating sequencing depth. **c** PLS-DA-plot of the two groups. **d** Taxa bar plot indicating the bacterial composition of each group in Phylum. **e** Firmicutes to Bacteroides (F/B) ratio of the two groups (*t*-test). **f** LEfSe analysis showing significantly different gut flora with a LDA score over 2 between the two groups

From the data of specimens collected at 5 months post-^131^I therapy, 2,253,269 high-quality sequences were used (average 60,832 per sample). The total number of ASV was 2,896 at 99% similarity level. The Good’s coverage of each group was over 99% (Fig. 5a). Rarefaction curve indicated good sequencing depths (Fig. 5b). No significant *α* diversity was noted between ER and NER groups. However, in line with the result of pre-^131^I therapy, a notable clustering effect of *β* diversity by response status post-^131^I was also revealed by PLS-DA-plot (Fig. 5c; R2X = 0.341, R2Y = 0.518, Q2Y =  − 0.083). Taxa bar plot showed elevation of Firmicutes and reduction of Bacteroidetes composition in the NER group (Fig. 5d), but no significant statistic difference was found in F/B ratio (Fig. 5e). LEfSe analysis illustrated significantly different bacterial taxa with a LDA score over 2.0 between ER and NER groups (Fig. 5f), including f_Erysipelotrichaceae, f_Lachnospiraceae, etc.

### Establishment of a predictive model consisting of gut microbiome and clinical data for response to ^131^I therapy

Based on the results above, we speculated that the gut microbiome signatures of ER or NER pre-^131^I therapy, combined with some clinical data possibly affecting ^131^I response, might construct a predictive model for response to ^131^I therapy in post-surgery patients with PTC. After screening and elimination of variables with multicollinearity, a multivariate analysis using binary logistic regression analysis was performed to analyze the remaining 14 variables to establish a predictive model. Finally, lymph node metastasis and relative abundance of g_Bifidobacterium and g_Dorea were finally chosen as the optimal set to establish the predictive model, with a *p* value of 0.003 and an overall percentage correct of 80.0% (Table 3), suggesting that this model performed well on predicting response to ^131^I therapy. High abundance of g_Bifidobacterium and g_Dorea and no lymph node metastasis predicted the patient to be ER, and vice versa. Of note, with a *p* value of 0.04, g_Dorea was an independent predictor of ^131^I therapy response.

**Table 3:** Summary of predictive model for response to ^131^I therapy by multivariate analysis using binary logistic regression

	Percentage correct	Chi-square	*p*	Variables	B	Wald	*p*
Model	80.0%	13.795	0.003	Lymph node metastasis	-1.658	2.832	0.092
				g_Bifidobacterium	33.229	2.774	0.096
				g_Dorea	161.779	4.206	0.040

## Discussion

Previous literatures have shown variable results in gut microbiome alterations induced by irradiation exposure [11, 16–18]. Nevertheless, the gut microbiota changes in the setting of ^131^I therapy have not been elucidated yet. Coinciding with some other findings in gut microbiota changes post radiation [19], our data illustrated that ^131^I therapy also resulted in deterioration of patients’ microbiome alpha diversity and alteration of microbial composition, with an enrichment of “harmful microbiota” and a reduction in “beneficial microbiota” on the whole. The impact of ^131^I therapy on gut microbiota was huge and durable, at least lasting until the end of our observation, 5 months post ^131^I administration. As shown by accumulating literatures, gut microbiota dysbiosis is involved in the development of a wide range of diseases, including diarrhea, allergy, cancer, aging, and diabetes [20]; thus, the dysbiosis after ^131^I therapy described in the present study has great influence on intestinal homeostasis and PTC patients’ health.

Among the changes of gut microbiome, alteration in F/B ratio is worth noting. In a healthy host, the majority of gut microbiome is typically dominated by four major phyla: Bacteroidetes, Firmicutes, Actinobacteria, and Proteobacteria [21]. Firmicutes and Bacteroidetes take up over 90% of the relative abundance of the gut microbiota, and their relationship plays a critical role in the maintenance of gut homeostasis. Aberrant ratio between the relative abundance of Firmicutes and Bacteroidetes (F/B ratio) has been found in a series of physiological and pathological conditions, including aging [22], tumor [23], obesity [24], type I diabetes [25], intestinal inflammation. In the present study, besides reduced F/B ratio, our data showed a prominent decline of Firmicutes post-^131^I therapy, which was in line with Firmicutes change after pelvic radiotherapy [17], indicating a comparable dysbiosis of gut microbiota after ^131^I therapy. Of note, F/B ratio was also found to be declined in NER group compared to ER group, indicating a possible connection between response to ^131^I therapy and microbial dysbiosis.

For the sake of understanding metabolic profile alterations of gut microbiota after ^131^I therapy, KEGG analysis of stool samples was performed. Some of the increases in metabolites after ^131^I therapy, such as bile acids and alanine, are in accordance with a previous reported characteristic elevation in radiation-induced acute intestinal symptoms in cervical cancer patients [26], indicating that metabolism of gut microbiome might share some common features post radiation, which warrants further study.

Numerous previous literatures have shown that gut microbiota exhibits value in predicting diseases [27, 28] and treatment response [29, 30]. In order to explore potential predictors of response to ^131^I therapy, gut microbiome differences were compared between ^131^I ER and NER groups both before and after the therapy. Different gut microbiota structures were identified as shown in LefSe analysis in Figs. 4f and 5f. Of note, in agreement with a previous study investigating gut microbiota signatures of distinct responses to neoadjuvant chemoradiotherapy [16], g_Dorea, a butyric acid-producing flora, regarded as an important component of functional microbiota in a healthy GI tract [31], was found to be significantly less abundant in ^131^I NER group pre-therapy in this study as well. Moreover, g_Dorea is later identified to be an independent predictor of response to ^131^I therapy, further suggesting its importance and predictive value. It is of interest that Erysipelotrichaceae, which belongs to the Firmicutes phylum, with multiple interactions with host immune response, gut inflammation, and lipid metabolism, etc. [32], was markedly decreased in NER patients in comparison with ER patients consistently before and after ^131^I therapy. Lachnospiraceae family, which is positively associated with protection against radiation-induced intestinal damages [33], was found to be remarkably increased in the NER group post-^131^I therapy, which awaits more study. The comparison of gut microbiota between groups with distinct responses to ^131^I may provide additional information on how the gut microbiome interacts with radiotherapy responses.

Moreover, a predictive model for response to ^131^I therapy of PTC patients was established in this study. Three variables, including lymph node metastasis, relative abundance of g_Bifidobacterium, and g_Dorea, for the first time, are identified to compose a model capable of predicting response to ^131^I therapy before the treatment initiation, which will be beneficial for customizing optimal therapeutic approaches for each PTC patient. The strategies to increase relative abundance of g_Dorea and g_Bifidobacterium in the gut microbiota may be promising in improving the response to ^131^I therapy in post-operative PTC patients. As stated before, g_Dorea, which produces butyric acid, is identified to be an independent predictor of response to ^131^I therapy in this study. Intriguingly, short-chain fatty acids (SCFAs), including butyric acid, are able to upregulate sodium/iodide (Na^+^/I^−^) symporter (NIS) expression through epigenetic modification, thus promote I^−^ uptake and beneficially affect response to ^131^I therapy[34], which may be one of the underlying mechanisms and needs more investigation.

To sum up, the present study demonstrates gut microbiota changes after ^131^I therapy in post-surgery PTC patients. Differences in gut flora are also found between ^131^I ER and NER groups both pre- and post-^131^I therapy, suggesting microbiome may be associated with therapeutic responses. Hence, a predictive model is established to provide a noninvasive tool for predicting response to ^131^I therapy prior to treatment initiation.

These conclusions are based on analysis of data obtained from a small sample pool, and reported in the pilot study herein. The long-term systemic effects of alterations in gut microbiome after ^131^I therapy still need rigorous evaluation. With the aim to restore a healthy intestinal microbial ecosystem, which is beneficial for the response to ^131^I therapy, personalized modulation of gut microbiota of PTC patients during the therapy, including dietary changes, administration of antibiotics or probiotics, and fecal microorganism transfers, is promising. Since advanced PTC patients with distant metastasis often have unfavorable prognosis, future studies on gut microbiota with larger cohort of these patients and employing whole genome metagenomics approaches should be carried out. It will shed new light on prediction and promotion of ER to ^131^I therapy in high-risk PTC patients. Mice model of ^131^I therapy is needed to further explore the complicated association between gut microbiome and response to ^131^I therapy.

## Data Availability

The datasets generated during and/or analyzed during the current study are available from the corresponding author on reasonable request.
